# Biological Assay to Determine Gonadotropin Potency: From In Vivo to In Vitro Sustainable Method

**DOI:** 10.3390/ijms24098040

**Published:** 2023-04-28

**Authors:** Francesco Nevelli, Angelo Palmese, Ralf Gleixner, Flavio Peroglio, Cosimo-Walter D’Acunto, Aurora Dadone, Thomas D’Hooghe, Monica Lispi

**Affiliations:** 1 Analytical Development Biotech—Global Analytical Development—Global Development & Launch—Global Healthcare Operation, Istituto di Ricerche Biomediche “Antoine Marxer” RBM S.p.A., Colleretto Giacosa, Via Ribes, 1, 10010 Samone, Italy; 2 Analytical Development Biotech—Global Analytical Development—Global Development & Launch—Global Healthcare Operation, Merck Serono S.p.A., Piazza del Pigneto, 9, 00176 Rome, Italy; 3 Ares Trading S.A., Rue de l’Ouriette 151, 1170 Aubonne, Switzerland; 4 GHO Ivrea QC & Scientific Excellence—Global Analytical Development—Global Development & Launch—Global Healthcare Operation, Istituto di Ricerche Biomediche “Antoine Marxer” RBM S.p.A., Colleretto Giacosa, Via Ribes, 1, 10010 Samone, Italy; 5 Global Medical Affairs Fertility, Merck Healthcare KGaA, Frankfurter Strasse 250, 64293 Darmstadt, Germany; 6 Department of Development and Regeneration, Laboratory of Endometrium, Endometriosis & Reproductive Medicine, KU Leuven, Oude Markt 13, 3000 Leuven, Belgium; 7 Department of Obstetrics, Gynecology, and Reproductive Sciences, Yale University Medical School, 333 Cedar St., New Haven, CT 06510, USA; 8 PhD School of Clinical and Experimental Medicine, Unit of Endocrinology, University of Modena and Reggio Emilia, Viale A. Allegri 9, 42121 Reggio Emilia, Italy

**Keywords:** r-hFSH, forced degradation, in vitro, in vivo, bioassay, potency, specific activity

## Abstract

Various preparations of follicle-stimulating hormone (FSH) are commercially available; however, they differ in glycoforms composition and purity owing to their respective sources. Additional chemical/physical changes can also be introduced during manufacturing and can impact their biological activity (biopotency), which is routinely assessed using an in vivo bioassay (Steelman–Pohley). This study aimed to determine whether an in vitro bioassay could assess biopotency by distinguishing between r-hFSH chemical/physical variants with similar ability to the in vivo bioassay. The specific activity (units of biological activity per mg of product) of variants of r-hFSH generated through enrichment (acidic/basic), stress (oxidative/acidic pH) and enzymatic treatment (desialylation and desialylation/degalactosylation) was compared using the in vivo and in vitro bioassays. The in vitro bioassay reliably detected potential chemical/physical modifications in r-hFSH variants that may impact biopotency. Overall, the methods demonstrated a comparable ability to detect changes in specific activities due to chemical/physical differences in r-hFSH variants. These data indicate that the in vitro bioassay is suitable to replace the in vivo bioassay.

## 1. Introduction

Follicle-stimulating hormone (FSH) induces follicular growth in humans: it regulates follicle recruitment, oocyte selection and sex steroid hormone synthesis, thus preparing the reproductive tract for fertilization, implantation and pregnancy [1]. By binding to, and subsequently activating, the FSH-receptor (FSHR), FSH regulates cellular metabolism and oocyte survival/maturation [2]. 

Various exogenous preparations of human FSH (hFSH) are commercially available, which are derived either from purified human urine (e.g., highly purified human menopausal gonadotropin [hMG-HP] or highly purified urinary FSH [u-hFSH-HP]) or obtained using recombinant DNA technology (r-hFSH) [3,4]. Follitropin alfa (r-hFSH; GONAL-f^®^, Merck Healthcare KGaA, Darmstadt, Germany [hereafter referred to as r-hFSH]) was the first r-hFSH produced using recombinant DNA technology [3,4]. The amino acid sequence of r-hFSH is identical to that of endogenous FSH [4], and comparable to endogenous FSH extracted from post-menopausal women in terms of pharmacokinetic (PK)/pharmacodynamic (PD) activity [5]. 

The hFSH products available for clinical use differ in terms of glycoform composition and purity, owing to their respective sources. Consequently, their PK and PD as well as their related clinical response differ among products [2]. Furthermore, the various exogenous preparations have also been shown to differ in terms of physicochemical characteristics, which result from post-translational modifications and changes incurred during production, such as oxidation, changes in pH during the manufacturing process or non-gonadotropin process residue [6]. Such variations may impact biopotency, which measures the biological activity of hFSH preparations [7,8,9]. 

Post-translational modifications, such as glycosylation (e.g., sialylation) and oxidation, play a crucial role in the stability and bioactivity of FSH preparations [10]. Glycosylation of the α subunit at both sites (Asn52 and Asn78) [11] is critical for normal biochemical functions [12]. The β subunit contains two glycosylation sites (βAsn7 and βAsn24), which affect metabolic clearance, FSHR binding and signal transduction [11,13]. In addition, the degree of terminal sialylation determines the charge of FSH and is the major factor governing the in vivo clearance rate. More acidic glycoforms have longer elimination rates due to reduced renal clearance, while less acidic glycoforms have a higher affinity for the FSHR [2]; however, even though acidic glycoforms are associated with higher metabolic stability and longer half-life, they show reduced biopotency and receptor binding activity [2,14]. Furthermore, while oxidation of methionine residues does not directly impact regions critical for FSHR binding, oxidation of these residues may lead to conformational changes that indirectly impact bioactivity, efficacy and safety. As such, analysis of post-translational modifications is common practice in characterization studies of biopharmaceuticals [10].

The Steelman–Pohley bioassay, a rat in vivo bioassay [15], is routinely used to assess r-hFSH biopotency by measuring ovarian weight increase [16], and is used to compare the biopotency of new r-hFSH test batches with that of a r-hFSH reference house standard (RHS) previously calibrated against the FSH international standard (IS) for quality control purposes [17]. The Steelman–Pohley bioassay is based on the linear relationship between the dose of FSH (in IU) administered and the increase in ovarian weight of immature rats versus a reference standard. Regulatory bodies require FSH biopotency to be quantified for quality control purposes, and currently the Steelman–Pohley bioassay is the only method published in the pharmacopoeias [18]; however, this bioassay has recognized disadvantages. Specifically, it is time-consuming as it necessitates the use of animals and, as such, relies on the development and health of the animals, with treatment alone occurring over 4 days [19]. Meanwhile, research has focused on the development of alternative, more sensitive in vitro bioassays to more accurately measure the biopotency of biological products for quality control purposes. 

The switch from the Steelman–Pohley to an in vitro bioassay for the assessment of FSH biopotency is attractive for several reasons. First, and most importantly, for ethical reasons—to replace animal use in quality control activity as recommended by 3R principles (Replacement, Reduction, Refinement) [20]. Secondly, to potentially reduce assay time, and reduce costs related to animal purchase and animal room maintenance. Lastly, since each animal represents a different organism and each animal responds differently to treatment, an in vitro bioassay may be expected to reduce this inherent inter-animal variability, allowing for a more precise determination of biopotency of a new product batch when compared to a standard, which is the actual scope of a product release test according to Quality Control standard requirements for biological products [7].

For this study, an in vitro bioassay was developed to determine biopotency by detecting r-hFSH dose-dependent increases in cAMP following FSH receptor activation, representing a potential alternative to the Steelman–Pohley bioassay [7,21]. Importantly, the developed in vitro bioassay and the currently used in vivo bioassay use different methodologies to determine FSH biopotency. The in vivo bioassay measures a clinically relevant endpoint (ovarian weight), which is influenced by various factors, including mechanism of action, route of administration, clearance and the animal model used; however, PK and PD from animal models may not provide an accurate prediction of PK, PD and clinical efficacy and safety for humans. In comparison, the developed in vitro bioassay evaluates the mechanism of action of FSH by measuring cyclic adenosine monophosphate (cAMP), which is catalyzed from adenosine triphosphate (ATP) by the enzyme adenylate cyclase after the binding of FSH to the FSHR [22]. Additionally, the developed in vitro bioassay and the currently used in vivo rat bioassay also differ with respect to biological assessment. For example, r-hFSH injected into rats displays a longer half-life for r-hFSH forms presenting high sialylation and antennarity grade, leading to higher biopotency in vivo (Steelman–Pohley) compared with low sialylated forms. In vitro, the concept of half-life of r-hFSH is not applicable as it is not possible to directly measure absorption, metabolism and clearance of FSH in the physiological environment of an in vitro bioassay. Here, we evaluated whether the in vitro bioassay could discriminate potential chemical and structural modifications to FSH molecules which may impact the absorption, metabolism and clearance of the drug in the physiological environment, in a similar manner to the in vivo bioassay. 

The aim of this study was to evaluate the suitability of a new in vitro bioassay, which was specifically developed to assess FSH biopotency for quality control purposes, and replace the currently used in vivo Steelman–Pohley bioassay described in EU Pharmacopoeia [15]. To achieve this, we assessed the ability of the in vitro bioassay compared with the currently used in vivo bioassay (Steelman–Pohley) to detect modifications in critical quality attributes generated through enrichment and enzymatic treatment in r-hFSH. Notably, for r-hFSH, the in vitro bioassay was recently approved by the European Medicines Agency (EMA) to replace the in vivo bioassay after obtaining a positive opinion from the Committee for Medicinal Products for Human use (CHMP) on 27 October 2022 for GONAL-f^®^ and Pergoveris^®^.

## 2. Results

FSH variants were generated through enrichment (acid species enrichment, basic species enrichment), stress (oxidative stress, acidic pH stress) and enzymatic treatment (desialylation and desialylation—degalactosylation) and the ability of the in vitro bioassay to detect modifications in critical quality attributes in each variant was then compared with the currently used in vivo bioassay (Steelman–Pohley).

### 2.1. Acid Enrichment

The impact of acid enrichment on physiochemical properties and biopotency was investigated in samples following charge-based fractionation.

#### 2.1.1. Dose–Response Curves

For acid enriched variants, as expected, the in vitro biopotency fell outside of the method linearity range (60–144%), which was determined during the method validation study, but no impact on the shape or behavior of the dose–response curve was observed (Supplementary Figure S1A). Similarly, no difference in the shape or behavior of the dose–response curve was observed between acid enriched variants and RHS with the in vivo bioassay (Supplementary Figure S1B).

#### 2.1.2. Physicochemical Characteristics

Acid enriched variants displayed increased sialylated glycans, with a corresponding reduction in glycans with exposed terminal galactose residues and increased antennarity (a consequence of the enrichment of more sialylated species) compared with untreated samples.

#### 2.1.3. Specific Activity

The in vitro bioassay detected a significant reduction in specific activity (Δ% −32% to −49%) as a result of acid enrichment compared with the untreated r-hFSH samples (*p* = 0.001) (Supplementary Figure S2A; Table 1). Conversely, the in vivo bioassay detected a significant increase in specific activity (Δ% +45% to +95%) as a result of acid enrichment compared with untreated r-hFSH samples (*p* = 0.008) (Supplementary Figure S2B; Table 1), indicating reduced clearance. 

Although the in vitro and in vivo bioassays exhibited opposite results with respect to specific activity, both methods were effective in their ability to detect physico-chemical modifications in r-hFSH resulting from acid enrichment.

### 2.2. Basic Enrichment

The impact on physicochemical characteristics and the biopotency of r-hFSH basic enrichment was assessed in samples following charge-based fractionation. 

#### 2.2.1. Dose–Response Curves

No impact on the shape or behavior of the dose–response curve was noted with either the in vitro or in vivo bioassays after basic enrichment, compared with the RHS (Supplementary Figure S3A,B).

#### 2.2.2. Physicochemical Characteristics

Basic enriched variants displayed lower levels of sialylation (and corresponding higher levels of terminal galactosylation) and a lower level of N-glycosylation site occupancy on the β subunit, compared with untreated samples. Additionally, simpler (mostly mono- and di-antennary) glycans were enriched in the basic fractions.

#### 2.2.3. Specific Activity

Following basic enrichment, a slight increase in specific activity was observed in two of the three samples tested using the in vitro bioassay (Δ% +16 and +20%, respectively) compared with untreated r-hFSH samples (*p* = 0.012) (Supplementary Figure S4A; Table 1). However, no variation in specific activity (Δ*% −*4%) was noted with basic enrichment of the third sample (FSE2004), indicating that this variant may contain other species that could negatively impact specific activity, neutralizing the desialylation effect (e.g., aggregates, free subunits and/or misfolding). As such, this sample was not included in the analysis. Conversely, a significant loss in specific activity was detected using the in vivo bioassay following basic enrichment (Δ% from *−*48% to *−*68%) compared with untreated r-hFSH samples (*p* = 0.002) (Supplementary Figure S4B; Table 1). 

Despite detecting an inverse correlation with respect to specific activity, both methods were effective for detecting physico-chemical modifications brought by basic enrichment; however, the magnitude of difference was greater for the in vivo bioassay than for the in vitro bioassay.

### 2.3. Oxidation

The impact of oxidation on physicochemical characteristics and biopotency was assessed in the samples following incubation with 0.5% H_2_O_2_ for 60 min. 

#### 2.3.1. Dose–Response Curves

In vitro biopotency fell outside of the linearity range (60–144%) determined in the method validation study for all analyses in response to oxidative stress. The very low sample potency led to parallelism failures for the dose–response curves compared with the RHS (Supplementary Figure S5A); however, no impact on the shape or behavior of the dose–response curve was noted for the oxidized samples analyzed using the in vivo bioassay (Supplementary Figure S5B). 

#### 2.3.2. Physicochemical Characteristics

Overall, the oxidized samples displayed higher levels of oxidation on α Met 29 (about 40%), α Met 47 (between 39% and 52%), α Met 71 (about 97%) and on β Met 109 (about 98%) compared with untreated samples.

#### 2.3.3. Specific Activity

Oxidative stress resulted in an almost complete loss of in vitro specific activity (Δ% from *−*73% to *−*82%; *p* < 0.001) (Supplementary Figure S6A; Table 1) and significantly decreased in vivo specific activity (Δ% from *−*9% to *−*16%; *p* = 0.006) (Supplementary Figure S6B; Table 1) compared with untreated r-hFSH samples.

Overall, both methods demonstrated an effective ability to detect oxidative stress; however, the in vitro method displayed greater sensitivity.

### 2.4. Acid pH Stress

The influence of acidic pH on the physicochemical properties of r-hFSH and its subsequent impact on biopotency was assessed in samples incubated with a 1:1 dilution of sodium citrate 0.5 M at pH 3.0 for 3 days. 

#### 2.4.1. Dose–Response Curves

In vitro biopotency fell outside of the linearity range (60–144%) determined in the method validation study for all analyses in response to acid pH stress (Supplementary Figure S7A); however, no impact on the shape or behavior of the dose–response curve was noted in acid pH stressed samples analyzed using the in vivo bioassay (Supplementary Figure S7B).

#### 2.4.2. Physicochemical Characteristics

Exposure to acidic pH stress resulted in a shift toward less sialylated forms at all glycosylation sites for all samples compared with untreated samples, although this was not statistically significant. This decrease in sialylation resulted in a subsequent increase in galactosylation. Additionally, an increased level of free FSH subunits was observed for all acid pH stressed variants compared with untreated samples.

#### 2.4.3. Specific Activity

After exposure to acid pH stress, a significant decrease in specific activity was noted using both the in vitro bioassay (Δ% from *−*79% to *−*83%; *p* < 0.001) (Supplementary Figure S8A; Table 1) and in vivo bioassay (Δ% from *−*62% to *−*70%; *p* < 0.001) (Supplementary Figure S8B; Table 1), compared with untreated r-hFSH samples.

Overall, both methods displayed a similar effective ability to detect changes resulting from acid pH stress.

### 2.5. Total Desialylation

The impact of total desialylation on the physicochemical properties and biopotency was assessed in samples incubated with sialidase A for 18 h.

#### 2.5.1. Dose–Response Curves

Totally desialylated FSH samples analyzed using the in vitro bioassay displayed differences in the shape of the dose–response curves (in particular in the low asymptote level), resulting in loss of parallelism with the RHS (Supplementary Figure S9A,B); however, no difference in the shape of the dose–response curves was observed with the totally desialylated samples analyzed using the in vivo bioassay (Supplementary Figure S9C). In vitro dose response was investigated to ensure that failure resulted from loss in sialylation rather than the presence of sialidase or its inhibitor in the samples. Indeed, additional samples were prepared as follows: the sialidase inhibitor was diluted in the sample at the same concentration as in the treated samples; then, the sialidase was added at the same concentration as in the previously treated samples. In this way, the sialidase was immediately blocked by its inhibitor, but it was possible to investigate the eventual impact of the enzyme and its inhibitor on the bioassay dose–response curve. The dose–response curves obtained with these samples showed good parallelism with the RHS concentration curve, demonstrating that loss in sialylation impacted on the shape of the dose–response curve.

#### 2.5.2. Physicochemical Characteristics

Total desialylation resulted in the almost complete hydrolysis of sialic acid at all *N*-glycosylation sites on both subunits: <1% of mono and di-sialylated residual glycoforms were detected on Asn52 of the α subunit and Asn7 of the β subunit, compared with untreated samples.

#### 2.5.3. Specific Activity

Totally desialylated samples analyzed by in vitro bioassay displayed significantly increased specific activity (Δ% from +90% to +115%) compared with untreated r-hFSH samples (*p* < 0.001) (Supplementary Figure S10A; Table 1). Conversely, the totally desialylated samples displayed significantly decreased specific activity using the in vivo bioassay (Δ% from −81% to *−*94%) compared with untreated r-hFSH samples (*p* < 0.001) (Supplementary Figure S10B; Table 1).

Despite detecting an inverse correlation with respect to specific activity, both methods showed effective ability in detecting physico-chemical modifications in r-hFSH resulting from total desialylation.

### 2.6. Different Levels of Sialylation

The impact of desialylation on the physicochemical properties and biopotency of r-hFSH was assessed in samples containing 100%, 75% and 50% desialylated r-hFSH.

#### 2.6.1. Dose–Response Curves

**Figure 1:**
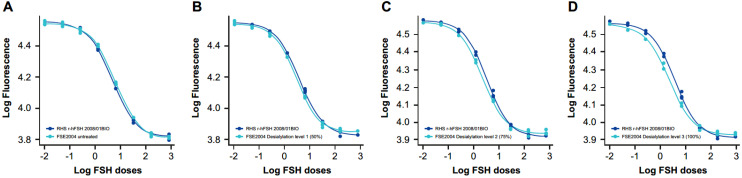
In vitro dose–response curves for RHS versus samples at different sialylation levels: (**A**) FSE2004 untreated, (**B**) FSE2004 desialylation level 1 (50%), (**C**) FSE2004 desialylation level 2 (75%) and (**D**) FSE2004 desialylation level 3 (100%). RHS (dark blue); FSE2004 desialylated sample (light blue).

**Figure 2:**
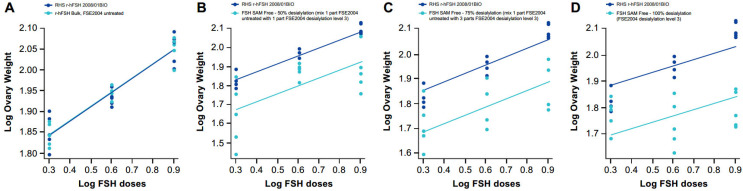
In vivo dose–response curves for RHS versus samples at different sialylation levels: (**A**) FSE2004 untreated, (**B**) FSE2004 desialylation level 1 (50%), (**C**) FSE2004 desialylation level 2 (75%) and (**D**) FSE2004 desialylation level 3 (100%). RHS (dark blue); FSE2004 desialylated sample (light blue).

Samples at different sialyation levels produced varying modifications in the shape of the dose–response curve in vitro that, in some cases, resulted in a loss of parallelism between the samples and the RHS (Figure 1 and Figure 2); however, a quantitative analysis was performed by calculating z-scores for the degree of sialylation in the samples. For this, samples of different levels of sialylation were generated (untreated, 50% [level 1], 75% [level 2] and 100% [level 3]) and analyzed by glycan mapping, as previously described. 

#### 2.6.2. Physicochemical Characteristics

As expected, the samples at different levels of sialylation displayed a lower level of sialylation compared with untreated samples. Using glycan mapping analysis, the measured Z numbers for the different samples decreased linearly with the spiking percentage (fully desialylated samples) and were lower than 160, as expected (of note, 160 is the Z number obtained for the enriched basic variants).

#### 2.6.3. Specific Activity

**Figure 3:**
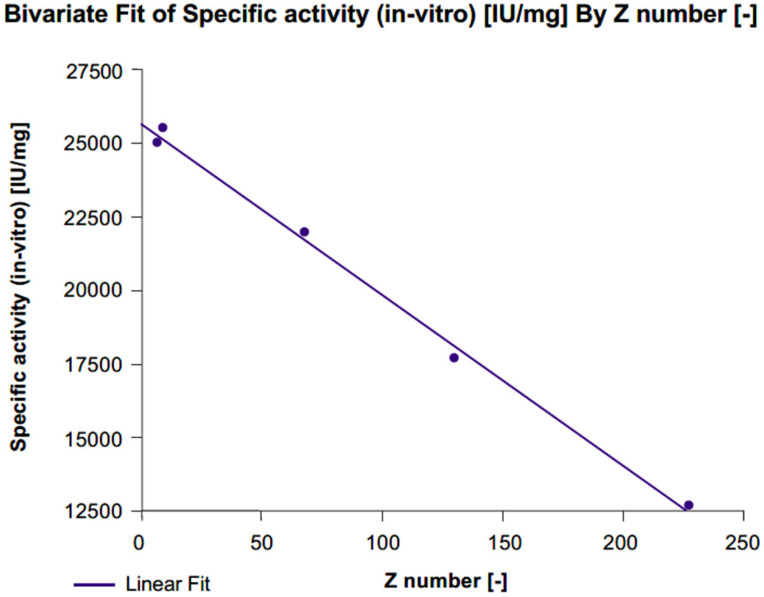
Correlation between z-score and in vitro specific activity for FSE2004 samples.

**Figure 4:**
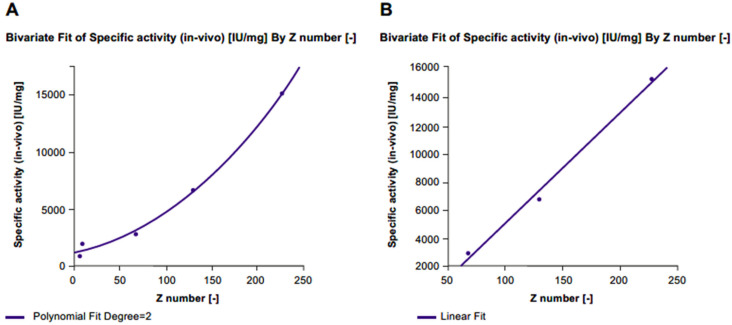
Correlation between z-score and in vivo specific activity for (**A**) all FSE2004 samples and (**B**) FSE2004 100% desialylated sample omitted.

In vitro specific activity significantly increased with each level of desialylation (level 1: Δ% +40%, level 2: Δ% +73%, level 3: Δ% +101%) compared with untreated r-hFSH samples (Figure 3; Table 1). A linear correlation between z-score and in vitro specific activity was confirmed by a highly significant fit line slope (*p* < 0.001; R^2^ = 0.996). Conversely, a gradual decrease in in vivo specific activity correlated with desialylation level (Level 1: Δ*% −*56%, level 2: Δ*% −*81%, level 3: Δ*% −*87%) compared with untreated r-hFSH samples (Table 1). The correlation between z-score and in vivo specific activity was well described by a second-degree polynomial equation (R^2^ = 0.995) due to the fact that after the de-sialylated level 2 (75%; z-score = 68), the correlation curve reached a plateau (Figure 4A). A linear correlation between z-score and in vivo specific activity was observed when 100% desialylated samples (desialylation level 3) were not included in the analysis. This was confirmed by an almost significant fit line slope (*p* = 0.0582 and an R^2^ = 0.992) (Figure 4B).

Both in vivo and in vitro bioassays showed a linear correlation between z-score (sialylation level) and sample specific activity; however, as expected, an inverse correlation was observed (in vitro biopotency increased while in vivo biopotency decreased).

### 2.7. Desialylation and Degalactosylation

The impact of desialylation and degalactosylation on the physicochemical properties and biopotency of r-hFSH was assessed in samples incubated with sialidase A for 18 h followed by treatment with galactosidase.

#### 2.7.1. Dose–Response Curves

Desialylation and degalactosylation resulted in significant modifications in the dose–response curves compared with RHS in both the in vitro and in vivo bioassays. This resulted in a loss of parallelism between the dose–response curves for the samples and the RHS (Supplementary Figure S11A,B).

#### 2.7.2. Physicochemical Characteristics

Desialylation and degalactosylation resulted in complete hydrolysis of sialic acid on all *N*-glycosylation sites of both subunits, with almost complete degalactosylation observed.

#### 2.7.3. Specific Activity

The in vitro bioassay detected a significant increase in specific activity as a result of desialylation and degalactosylation (Δ% from +36% to +64%) compared with untreated r-hFSH samples (*p* = 0.001) (Figure S12A; Table 1). Conversely, the in vivo bioassay detected a complete loss of specific activity following desialylation and degalactosylation (Δ% from −80% to −95%) compared with untreated samples (*p* < 0.001) (Figure S12B; Table 1).

The two methods displayed effective ability in detecting differences in biopotency due to desialylation and degalactosylation (as demonstrated by Δ%); however, an inverse correlation was observed (in vitro biopotency increased while in vivo biopotency decreased).

## 3. Discussion

The results of this study show that the in vitro bioassay can accurately and reliably identify potential chemical or structural changes in FSH molecules that may impact biopotency. Additionally, analysis of r-hFSH variants, generated through enrichment, stress or enzymatic treatment, also allowed us to gain insights into the relationship between r-hFSH sialylation and biological activity.

The presence or absence of post-translational modifications, and/or changes in their relative abundance, may impact clinical efficacy and safety; therefore, analysis of post-translational modifications is common practice in characterization studies of biopharmaceuticals [10]. Overall, our data show that both the in vitro and in vivo bioassays demonstrated effectiveness in identifying differences in critical quality attribute levels (sialylation, oxidation, free-subunits) between r-hFSH variants. However, although an inverse correlation with respect to dose–response curves and specific activity were detected under certain conditions, as expected, the percentage difference (Δ%) in specific activity between FSH variants and untreated samples—and therefore the ability to detect an effect rather than the direction of the effect—was successfully used to compare the two methods.

This study showed that forced degraded r-hFSH, along with charge and glycosylation variants, displayed modifications in critical quality attributes compared with untreated r-hFSH samples, with sensitivity and direction of effect on specific activity varying depending on the type of assay used. Overall, both the in vivo and in vitro bioassays detected an increase in free subunits, which resulted in significantly reduced r-hFSH specific activity compared with untreated samples (*p* < 0.001 for both). Both bioassays also detected an increased presence of r-hFSH oxidized forms (up to 50% above product specification), which correlated with significantly reduced r-hFSH specific activity in vitro (*p* < 0.001) and, to a lesser extent, in vivo (*p* = 0.006). A gradual decrease in r-hFSH sialylation reduced r-hFSH-specific activity in vivo, indicating a second-order polynomial correlation (R^2^ = 0.995), but increased r-hFSH-specific activity in vitro, signifying a negative linear correlation (R^2^ = 0.996). Additionally, r-hFSH variants with higher levels of sialylation, such as those exposed to acid enrichment, displayed decreased specific activity in vitro and increased specific activity in vivo, whereas r-hFSH variants with lower levels of sialylation and antennarity, such as totally desialylated variants or those exposed to basic enrichment, displayed increased specific activity in vitro and decreased specific activity in vivo. Conversely, although variants exposed to acid pH displayed lower levels of sialyation, both bioassays detected a significant reduction in specific activity. Lastly, total desialylation doubled the in vitro specific activity compared with untreated samples (*p* = 0.000), whereas total desialylation resulted in an almost complete loss of in vivo specific activity (*p* = 0.000). 

The results of this study are in line with previous studies reporting the impact of modifications in critical quality attributes on FSH biopotency. For example, studies have shown that oxidative stress causes conformational changes in r-hFSH, leading to reduced biological activity [23], which is in line with the results reported here. Additionally, FSH variants with a high degree of sialylation and increased antennarity exhibit improved metabolic stability and increased half-life [2]; however, while highly acidic glycoforms of FSH have substantially longer half-lives, they may display decreased affinity for the FSHR, subsequently reducing their capacity to trigger cellular responses, such as the stimulation of cAMP and the production of estradiol [8,11,24,25,26]. Conversely, desialylated FSH glycoforms, or those with lower levels of sialylated glycans, with decreased antennarity display low biopotency, presumably due to the removal of sialic acid, which enhances FSH clearance [2,26,27]. Importantly, an increased di-antennarity at Asn52, in comparison with untreated samples, was observed with a basic enrichment procedure. This increase is commonly associated with increased FSHR affinity and more potent receptor activation [8,11,24]. 

The impact of these changes on specific activity differed between the in vitro and in vivo bioassays, with an opposite direction of effects observed. These differences may be explained by the fact that the clearance rate contributes to the effects measured in vivo, but these are not taken into account with in vitro bioassays [8,24,28]. However, differences could also be due to suboptimal endpoint selection, with biological activity in vivo potentially varying depending on the physiological endpoint and animal model chosen (e.g., ovarian weight vs. estradiol production as a way to evaluate FSH effect on ovarian response) [8]. 

Besides effectively discriminating between different variants of FSH, the in vitro bioassay is favored over the in vivo bioassay for several other reasons. Firstly, and most importantly, regulatory bodies have long encouraged the development of alternatives to animal testing. The in vivo bioassay is dependent on the use of animals and is also time-consuming and reliant on scheduling, logistics and availability of services. Secondly, the in vivo bioassay in rats is highly dependent on rat PK and PD, but concerns have been raised over the validity of comparing the PK and PD of rats with those of humans [29], particularly human recombinant glycoproteins. However, as shown by the data reported here, there are potential advantages to performing comparisons in rats where inverse effects are observed. Thirdly, despite continued improvements in accuracy and reliability gained from years of experience with in vivo bioassays, the limits of bioactivity defined by the pharmacopeia imply that there may be a loss of accuracy when a limited number of in vivo bioassays are performed, despite being calibrated against an IS [28]. As well as eliminating the need for animals, the in vitro bioassay can be expected to allow for the rapid analysis of a large number of samples. Thus, the newly developed in vitro bioassay may provide a viable and ethical alternative to the in vivo bioassay for quality control purposes. This is important as quality control is an essential part of the pharmaceutical quality system and can be implemented throughout the different stages of a product lifecycle to enhance the quality and availability of medicines around the world, which is in the interest of public health. The implementation of a quality system allows a control process for performance and product quality to be established and maintained, thereby ensuring continued suitability and capability of processes [30].

The in vitro bioassay also demonstrated the ability to distinguish between different variants of FSH, ensuring the release of FSH batches with consistent performance and precision, proving a reliable replacement for the current in vivo bioassay. Furthermore, owing to the process of RHS calibration mentioned previously, biopotency quantification remains unaffected by a potential switch from an in vivo to an in vitro method, as both methods measure FSH biopotency in IU. With r-hFSH labelled in both the µg of r-hFSH protein and in IU, as required by the health regulatory authorities [19], the adoption of an in vitro bioassay for the determination of FSH biopotency is a unique opportunity that will continue to provide clinicians with confidence that they are using a product that has been manufactured to the highest standards that delivers a precise dose of FSH with consistent results [19,31]. 

Although these results demonstrate the capability of the in vitro bioassay to replace the in vivo bioassay, we recognize that forced degraded samples and enriched variants of r-hFSH may contain more than one structural/chemical modification; therefore, the effect of discrete modifications could not be studied. Furthermore, the potency measurements for the in vitro and in vivo bioassays are only calibrated against the specific post-translational modifications present in IS 08/282. However, when the in vitro bioassay is combined with the evaluation of other critical quality attributes, such as dissociated free subunits, oxidized forms and glycans, the resulting analysis can be considered fully representative of current in vivo testing, since no other critical quality attributes impact biological activity. Finally, although the in vitro bioassay directly evaluates the main mechanism of FSH action—by measuring cAMP levels—other signaling pathways that modulate FSH activity, such as extracellular signal-regulated kinase 1/2 (ERK1/2) and p38 mitogen-activated protein kinase (p38MAPK), were not considered during this study. However, it is important to note that targeting the main signaling pathway using a fixed exposure time and a fixed dose–stimulation curve is considered the standard approach for the development of novel bioassays that can accurately measure r-hFSH biopotency for quality control purposes, according to US Pharmacopoeia [28,32]. Additionally, the broad range of r-hFSH concentrations used to establish the dose–simulation curve (4-PL curve) used in this study allowed for the full characterization of the relationship between FSH dose and cell signaling response—from the absence of signaling due to low r-hFSH dose to saturated cell signaling response due to high doses of r-hFSH. Lastly, the in vitro bioassay was validated according to linearity, relative accuracy and intermediate precision, as recommended by the US Pharmacopeia [33].

## 4. Materials and Methods

### 4.1. Samples

Three batches of r-hFSH serum-free DS (FSE2003, FSE2004 and FSE2005) were available for testing. All batches of r-hFSH were provided by Merck S.L. Tres Cantos, Madrid, Spain, an affiliate of Merck KGaA (Darmstadt, Germany), and the currently used RHS (RHS r-hFSH 2008/01BIO) was provided by Merck Healthcare KGaA, Guidonia, Italy.

### 4.2. Materials

The HEK-293 cells transfected with FSHR and a coupled G protein were purchased from Merck Millipore (Milford, MA, USA). The GS HiRange kit was purchased from PerkinElmer Corp (Waltham, MA, USA). The Waters Xevo G2-XS Q-TOF mass spectrometer, equipped with an Acquity UPLC system, Acquity glycan BEH amide UPLC column and HPLC Alliance 2690 were purchased from Waters (Milford, MA, USA). The BIOSEP SEC-S2000 column was purchased from Tosoh Bioscience GmbH (Stuttgart, Germany). Amicon Ultra 3K centrifugal filters were purchased from Merck Millipore (Watford, UK). Water used in experiments was purified with a Milli-Q system from Merck Millipore (Milford, MA, USA). GlycoClean S cartridges were purchased from Prozyme Inc. (Hayward, CA, USA). The cell counter was purchased from Chemometec (Allerod, Denmark) and the homogeneous time-resolved fluorescence (HTRF) plate reader was purchased from Agilent (Santa Clara, CA, USA).

### 4.3. Forced Degraded and Molecular Variants

Variants were generated from three batches of r-hFSH (FSE2003, FSE2004, FSE2005) through enrichment (acid species enrichment, basic species enrichment), stress (oxidative stress, acidic pH stress) and enzymatic treatment (desialylation and desialylation—degalactosylation). The ability of the in vitro bioassay to detect modifications in critical quality attributes was compared with the currently used in vivo bioassay (Steelman–Pohley). The methods for the generation of each forced degraded r-hFSH sample and molecule variants (charge and glycosylation variants) are described in Table 2.

In addition, three samples of FSE2004 were incubated with sialidase, and these fully desialylated samples were mixed with intact samples to obtain different levels of sialylation: FSE2004 desialylation level 3 (100%); FSE2004 desialylation level 2 (75%)—obtained by mixing 1 part of untreated FSE2004 with 3 parts of FSE2004 desialylation level 3; and FSE2004 desialylation level 1 (50%)—obtained by mixing 1 part of untreated FSE2004 with 1 part of FSE2004 desialylation level 3.

After treatment, the samples were split into aliquots and stored at −20 ± 5 °C.

### 4.4. Determination of Protein Content

The protein content of each sample was determined using size-exclusion–high-performance liquid chromatography (SE–HPLC). To determine content, the r-hFSH samples were diluted in Kolliphor 188 buffer and analyzed by HPLC Alliance 2690 (Waters, Milford, MA, USA) using a BIOSEP SEC-S2000 column (Tosoh Bioscience GmbH, Stuttgart, Germany) at room temperature. Elution was carried out using a buffer containing 0.1 M sodium phosphate and 0.1 M sodium sulfate at pH 6.7, at a flow rate of 1 mL/min. Detection was carried out at 214 nm and protein content was determined by calibration against the RHS.

### 4.5. Determination of FSH Potency

#### 4.5.1. In Vitro Bioassay to Assess r-hFSH Biopotency

The in vitro bioassay used HEK-293 cells transfected with FSHR and a coupled G protein (Merck Millipore, MA, USA). The HEK-293 cell line was selected because it stably expresses human FSHR, allowing for the evaluation of hormone receptor binding affinity and activation, and has been shown to produce cAMP in response to FSH stimulation [34,35]. HEK-293 is qualified for such use within a defined working window; to ensure method suitability, test acceptance criteria are maintained, and each cell bank is tested for functionality at the beginning, middle and end of the defined working window [7,36].

Cells were detached from the flask and seeded into each well of a 96-well plate with r-hFSH (control sample, RHS and test samples). Cells were then stimulated with a fixed dose of phosphodiesterase (PDE) inhibitor to block cAMP metabolism and eight different doses of the RHS and r-hFSH test batch were prepared by serial dilution. After stimulation, the plate was incubated in a 5% CO_2_ incubator at 37 °C at target point.

Intracellular cAMP was quantified using the GS HiRange kit (PerkinElmer Corp, Waltham, MA, USA), which uses fluorescent resonance energy transfer (FRET) technology to detect cAMP. The GS HiRange kit consists of a lysing solution, cAMP labelled with a d2 fluorophore and an anti-cAMP antibody labelled with an EU3+ cryptate fluorophore. Cells were lysed using the lysis solution to release free cAMP into solution, and fluorimetry was used to quantify cAMP: excitation 330 nm/emission 620 nm and excitation 360 nm/emission 665 nm.

Each sample batch was tested in three independent runs, wherein each run was performed with independent cell cultures (i.e., derived from different flasks). For each run, data analysis was performed using parallel-line analysis (PLA) 3.0 software: the dose–response curves of the sample and RHS were interpolated with a four-parameter equation and the relative potency was calculated as the ratio of the inflection point of sample and RHS.

For the dose–response curves, parallelism was used to estimate the similarity between the sample being tested and the standard curve. A failure in the parallelism suitability test indicated that the tested sample and standard do not behave in a similar manner. Loss of parallelism impacts the reliability of the potency estimation; however, quantitative analysis may still be performed, in particular when sample potency resulted outside of the validated method linearity range (60–144% of the RHS potency).

#### 4.5.2. In Vivo Bioassay to Assess r-hFSH Biopotency

The biopotency of the r-hFSH samples was estimated using the Steelman–Pohley in vivo bioassay, performed according to both the published method [16] and the EP Follitropin 01/2020-2285-2286. For this, female Sprague Dawley rats aged 21–22 days, with body weight differing by no more than 10 g between the heaviest and the lightest rat (Charles River, Calco, Italy), were used. The animals were treated with human chorionic gonadotropin (hCG) and r-hFSH RHS, previously calibrated against the IS, or r-hFSH test batch for 3 consecutive days, and five subcutaneous injections were performed using doses of 2.0, 4.0 or 8.0 IU/rat (total of 3 mL/animal).

Rats were euthanized 72 h after the first injection; the method used for euthanasia was in agreement with the Italian D.Lvo No. 26 of 4 March 2014 (Authorization released by Italian Health Authority n° 234/2020-PR). The ovaries were removed and weighed, and ovarian weight was compared between animals treated with the r-hFSH RHS and those treated with the r-hFSH test batch by performing PLA using PLA 3.0 statistical software (Stegmann Systems, Rodgau, Germany).

Each sample batch was tested in two independent runs. For each run, data analysis was performed using PLA 3.0 software: the dose–response curves of the sample and RHS were interpolated with a linear equation and the relative potency was calculated as the distance of the sample and standard lines on the horizontal axes.

For the duration of the experiment, rats were housed in rooms with limited access (barriered rodent facility) in polycarbonate cages with stainless steel mesh tops and dust-free soft wood chip bedding. For environmental enrichment, paper bags containing dedusted wood chips were placed inside cages, along with a “rat house” and cotton rolls. HVAC systems ensured that temperature and relative humidity were maintained according to current legislation, i.e., 22 °C ± 2 °C and 55% ± 10%, respectively, with 15–20 air changes per hour (HEPA filtered). The rooms were illuminated by artificial lighting with a circadian cycle of 12 h of light (07:00–19:00).

Throughout the experiment, the health status of the rats was assessed at least twice daily. A pelleted diet coded “4RF 25 GLP Top Certificate”, containing adequate amounts of vitamins and trace elements, was used to feed the animals. The diet was available ad libitum to all animals, with additional pulverized food provided to facilitate food intake if needed. Drinking water, offered ad libitum in bottles to all animals, was supplied from the municipal water mains. The water and the diet used are analyzed twice per year for microbiological count, for the presence of heavy metals, other contaminants (e.g., solvents and pesticides) and other physical and chemical properties.

No ethics committee approval was needed for this study as the in vivo bioassay is required by European Pharmacopoeia [15] to assess the quality control of r-hFSH batches for market release; however, specific authorization for characterization of r-hFSH variants was provided by the Italian Minister of Health (Authorization n° 234/2020-PR (protocol 18ECB.11)). The approval of animals used, housing and welfare were guaranteed according to the Italian D.Lvo No. 26 of 4 March 2014. Physical facilities for accommodation and care of animals are in accordance with the provisions of the Italian D.Lvo 2014/26 and of Directive 2010/63/EU. The institute is fully authorized by the Italian Ministry of Health.

### 4.6. Calibration of Reference House Standard for the In Vitro Bioassay

The in vitro bioassay was used to determine FSH potency, which was assessed against a reference standard. The currently used RHS (r-hFSH 2008/01BIO), a GONAL-f^®^ 150 IU monodose filled by mass (11 μg) and already calibrated for the in vivo bioassay, was also calibrated against the IS (IS 08/282) in vitro in 15 analytical sessions. For this, the RHS and IS were diluted down to the first cell stimulation dose (816.0 ng/mL) based on the protein content of each vial (mg/container), which was determined by HPLC. The RHS and IS doses were administered at the same dose as the r-hFSH test batches. A percentage potency, relative to the IS, was calculated for each of the 15 analytical sessions and the mean was calculated. To be considered aligned, the mean percentage relative potency, measured by comparing the concentration–response curves of a manufactured test batch of RHS with that of IS, should fall between 88 and 112%. The acceptance criteria range was determined based on the precision acceptance criteria set for the validation study (12%) considering 100% as the target result. Since the mean percentage relative potency of RHS was 102%, it was considered aligned with the IS. To determine specific activity (IU/μg), the following equation was used:

RHS r-hFSH 2008/01BIO IU/container=126 IU ∗12 μg∗(120/100)=179.3 IU/container8.6 μg

where 126 IU is the IU of IS 08/282, 8.6 μg is the IS 08/282 protein content determined by HPLC, 12 μg is the RHS protein content determined by HPLC, 102 is the mean percentage relative potency of RHS and 100 is the target percentage. The ratio between the RHS measured IU per container and the μg protein content per container represents the RHS-specific activity, which corresponds to 14.94 IU/μg. As a result, the RHS was used in this study to calculate, for each batch, the IU content and the final relative potency value. Based on the process described above for RHS calibration, biopotency quantification required for quality control purposes may not be affected by a potential switch from an in vivo to an in vitro method, specific to the post-translational modifications present in IS 08/282. In particular, the declared IU content would not be modified even if quantified by the proposed in vitro bioassay.

### 4.7. Glycopeptide Mapping

For each sample, 200 μg of protein was denatured using 8M Guanidine, 130 mM Tris, 1 mM ethylenediaminetetraacetic acid (EDTA) pH 7.6. Samples were reduced by adding 20 μL dithiolthreitol (DTT) 500 mM (1 h, 37 °C) and then alkylated with 40 μL of iodoacetamide (IAM) 500 mM (30 min in the dark). Each sample was then washed with 200 μL of 2 M Urea, 50 mM Tris pH 8.0 on Amicon Ultra 3K cartridges. Washed samples were subjected to enzymatic hydrolysis with 10 μL of chymotrypsin (enzyme to substrate ratio 1:20, 4h, 37 °C). The chymotrypsin-digested samples were analyzed using ultra-performance liquid chromatography–electrospray tandem mass spectrometry (UPLC–ESI–MS/MS), on a Waters Xevo G2-XS Q-TOF mass spectrometer in MS^E^ mode equipped with an Acquity UPLC system (Waters, Milford, MA, USA), by injecting 25 µL using the autodilution mode starting from 10 µL of each sample in each vial. The peptides were separated on an Acquity glycan BEH amide UPLC column (1.7 μm, 2.1 × 150 mm) (Waters, Milford, MA, USA) and eluted at a flow rate of 0.2 mL/min, keeping the column at 50 °C with a mixture of 0.1% trifluoroacetic acid (TFA) in water (solution A) and 0.1% TFA in acetonitrile (CAN) (solution B), employing a gradient starting at 90% solution B. The mass spectrometer was operated in MS^E^ mode with the following parameters: capillary voltage 3 kV, sampling cone 30 V, source temperature 120 °C, desolvation temperature 300 °C, cone gas flow 50 L/H, desolvation gas flow 800 L/H and scan range 100–2500 *m*/*z*. The data were processed using Expressionist 13.0 software (Genedata, Basel, Switzerland).

### 4.8. Peptide Mapping

Sample proteins (100 µg) were denatured using 8M Guanidine, 1 mM EDTA and 130 mM Tris pH 7.6. Samples were reduced with 10 μL DTT 200 mM (1 h, 37 °C) and then alkylated with 25 μL IAM 200 mM (1 h, room temperature, in the dark). Each sample was then washed with 300 μL 6 M Urea, 100 mM Tris pH 8.0, on Amicon Ultra 3K cartridges (final volume 100 uL). Next, 200 μL of 50 mM Tris pH 8.0 was added to washed samples for enzymatic hydrolysis with Trypsin (enzyme to substrate ratio 1:17, 2 h, 37 °C). Samples were then treated with *N*-glycanase (18 h, 37 °C) to hydrolyze the *N*-glycosydic linkages between the glycans and the protein, to determine the *N*-glycosylation site occupancy. Each sample was analyzed by UPLC–ESI–MS/MS on a Waters Xevo G2-XS Q-TOF mass spectrometer in MS^E^ mode, injecting approximately 1 µg. Peptides were eluted at a flow rate of 0.4 mL/min, keeping the column at 60 °C, with a mixture of 0.1% FA in water (solution A) and 0.1% FA in ACN (solution B), employing a gradient starting at 99% solution A. Initial conditions were held for 2 min followed by a 50 min gradient in which the proportion of solution B was increased to 80%. The column was then washed for 3 min with 80% solution B and then re-equilibrated for 10 min under the initial conditions. The mass spectrometer was operated with the following parameters: capillary voltage 3 kV, sampling cone 30 V, source temperature 100 °C, desolvation temperature 250 °C, cone gas flow 20 L/H, desolvation gas flow 800 L/H and scan range 50–2000 *m*/*z*. The data were processed using Waters Biopharma Lynx 1.3.4 software (Milford, MA, USA).

### 4.9. Glycan Mapping

For glycan mapping, 250 μg of sample protein was denatured with 1% sodium dodecyl sulfate (SDS) and 143 mM 2-mercaptoethanol and then incubated with *N*-glycanase (24 h, 37 °C). The protein fraction was precipitated with ethanol, and the glycan fraction was collected, lyophilized and labelled with 2-aminobenzamide (2-AB) (30 min, 65 °C). After labelling, oligosaccharides were purified using GlycoClean S cartridge (Prozyme Inc., Hayward, CA, USA) and analyzed by reverse-phase HPLC (RP-HPLC) (Waters, Milford, MA, USA) with a GlycoSep C GKI-4721 7.5 × 7.5 mm column using a gradient of 500 mM ammonium acetate, pH 4.5 (eluent A) and 100% ACN (eluent B). The 2-AB labelled glycans were analyzed by fluorimetry (excitation wavelength 330 nm; emission wavelength 420 nm).

### 4.10. Data Analysis

#### 4.10.1. Potency of Treated and Untreated Samples Obtained by In Vivo and In Vitro Bioassays

For both the in vitro and in vivo bioassays, samples were tested during repeated independent analytical sessions (two sessions for the in vivo bioassay and three sessions for the in vitro bioassay). For each session, the sample relative potency was calculated using the PLA 3.0 statistical software (Stegmann Systems, Rodgau, Germany). The arithmetical mean between analytical sessions was then calculated to generate the mean potency value (%).

#### 4.10.2. In Vitro Bioassay

Once the mean potency values (%) had been generated for each test sample (treated and untreated) for the in vitro bioassay, the sample potency was calculated with respect to the RHS using the following equation:

Calculated sample potency (IU/mL) = RHS specific activity (IU/μg) × mean potency % × content (μg/mL)

where the specific activity of RHS was 14.94 IU/μg.

#### 4.10.3. In Vivo Bioassay

Once the mean potency value (%) had been generated for each test sample (treated and untreated) for the in vivo bioassay, the sample potency was calculated using the following equation:

Calculated sample potency (IU/mL) = mean potency % × nominal sample potency (IU/mL)

where the nominal sample potency corresponds to the IU/mL defined as target.

#### 4.10.4. Comparison between In Vivo and In Vitro Specific Activity of Treated and Untreated Samples

For each (treated and untreated) sample, the IU/mL (potency) was divided by the mg/mL concentration to determine specific activity using the following formula:

Specific activity (IU/mg) = Calculated sample potency (IU/mL)/protein content by HPLC (mg/mL)

The percentage difference (Δ%) in specific activity between the untreated and treated samples was calculated to evaluate whether the two methods could discriminate between different FSH variants related to differences in their chemical/physical structure. The effective ability of the in vitro and in vivo bioassays to detect variations in r-hFSH was assessed rather than the direction of the effect. An analysis of variance (ANOVA) test at the 95% level of confidence was performed to evaluate whether the calculated difference in specific activity was significant (*p* ≤ 0.005).

## 5. Conclusions

Chemical and/or structural modifications of r-hFSH strongly impact r-hFSH biopotency as measured by in vitro or in vivo bioassays. In most cases, both in vitro and in vivo bioassay methods demonstrated the ability to effectively detect differences in bioactivity caused by changes in critical quality attribute levels *(*sialylation, oxidation, dissociated subunits). The development of an in vitro bioassay for the accurate measurement of r-hFSH biopotency may serve to replace the in vivo bioassay, according to the 3R principles (Reduce, Replace, Refine), for the critical quality attributes analyzed. As one final concluding point, for r-hFSH, the in vitro bioassay was recently approved by the EMA to replace the in vivo bioassay after obtaining a positive opinion from the CHMP on 27 October 2022 for GONAL-f^®^ and Pergoveris^®^.

## Figures and Tables

**Table 1:** In vitro and in vivo results for each variant.

Category	Sample	In Vitro Activity (IU/mg) *	Δ% In Vitro	In Vivo Activity (IU/mg) ^†^	Δ% In Vivo	Z-Number
**Acid enriched**	**FSE2003 untreated**	11,858		14,316		226
	**FSE2003 acid enriched**	7153	−40%	20,785	+45%	294
	**FSE2004 untreated**	12,688		15,139		221
	**FSE2004 acid enriched**	6442	−49%	23,580	+56%	289
	**FSE2005 untreated**	11,804		14,038		221
	**FSE2005 acid enriched**	8011	−32%	27,414	+95%	289
**Basic enriched**	**FSE2003 untreated**	11,858		14,316		226
	**FSE2003 basic enriched**	14,219	+20%	7467	−48%	167
	**FSE2004 untreated**	12,688		15,139		221
	**FSE2004 basic enriched**	12,230	−4%	7912	−48%	169
	**FSE2005 untreated**	11,804		14,038		221
	**FSE2005 basic enriched**	13,744	+16%	4434	−68%	165
**Oxidized**	**FSE2003 untreated**	11,858		14,316		··
	**FSE2003 oxidized**	2963	−75%	12,717	−11%	··
	**FSE2004 untreated**	12,688		15,139		··
	**FSE2004 oxidized**	2222	−82%	12,764	−16%	··
	**FSE2005 untreated**	11,804		14,038		··
	**FSE2005 oxidized**	3172	−73%	12,771	−9%	··
**Acid pH**	**FSE2003 untreated**	11,858		14,316		··
	**FSE2003 acid pH**	2444	−79%	5383	−62%	··
	**FSE2004 untreated**	12,688		15,139		··
	**FSE2004 acid pH**	2168	−83%	4573	−70%	··
	**FSE2005 untreated**	11,804		14,038		··
	**FSE2005 acid pH**	2165	−82%	4576	−67%	··
**Total desialylation**	**FSE2003 untreated**	11,858		14,316		226
	**FSE2003 desialyl**	23,241	+96%	849	−94%	6
	**FSE2004 untreated**	12,688		15,139		221
	**FSE2004 desialyl**	25,058	+97%	843	−94%	7
	**FSE2005 untreated**	11,804		14,038		221
	**FSE2005 desialyl**	25,296	+114%	1900	−86%	7
	**FSE2012 untreated**	12,177		15,689		
	**FSE2012 desialyl T1**	23,193	+90%	3932	−81%	13
	**FSE2012 desialyl T2**	23,571	+94%	2960	−85%	12
	**FSE2012 desialyl T3**	25,545	+110%	3735	−82%	11
	**FSE2012 desialyl T4**	26,207	+115%	2915	−86%	7
**Different sialylation levels**	**FSE2004 untreated**	12,688		15,139		227
	**Desialylation level 1 (50%)**	17,723	+40%	6617	−56%	130
	**Desialylation level 2 (75%)**	21,963	+73%	2819	−81%	68
	**Desialylation level 3 (100%)**	25,530	+101%	1925	−87%	9
	**FSE2004 desialylated**	25,058	+97%	843	−94%	7
**Desialylated and degalactosylated**	**FSE2003 untreated**	11,858		14,316		226
	**FSE2003 Desial/Degal**	17,709	+49%	1324	−91%	6
	**FSE2004 untreated**	12,688		15,139		227
	**FSE2004 Desial/Degal**	17,317	+36%	743	−95%	6
	**FSE2005 untreated**	11,804		14,038		224
	**FSE2005 Desial/Degal**	19,379	+64%	2788	-80%	7

* Activity measured using the developed in vitro bioassay. **^†^** Activity measured using the Steelman–Pohley in vivo bioassay.

**Table 2:** Forced degradation and deglycosylation methods.

Category	Procedure
Acidic variant	Acid fractionation performed by manufacturing site.
Basic variant	Basic fractionation performed by CRO.
Oxidation	Incubation at room temperature with 0.5% H_2_O_2_ for 60 min.
Acidic pH	1:1 dilution with sodium citrate 0.5 M pH 3.0 and incubation with sialidase at 25 °C for 3 days.
Glycosylation (desialylated sample)	Incubation at 37 °C for 18 h with Sialidase A. The enzymatic activity was stopped by freezing at −20 ± 5 °C.
Glycosylation (desialylated + degalactosylated sample)	Incubation at +37 °C for 18 h with Sialidase A, followed by galactosidase. The enzymatic activity was stopped by freezing at −20 ± 5 °C.

## Data Availability

Any requests for data by qualified scientific and medical researchers for legitimate research purposes will be subject to Merck KGaA’s Data Sharing Policy. All requests should be submitted in writing to Merck KGaA’s data sharing portal (https://www.merckgroup.com/en/research/our-approach-to-research-and-development/healthcare/clinical-trials/commitment-responsible-data-sharing.html, accessed on 4 April 2023). When Merck KGaA has a co-research, co-development or co-marketing or co-promotion agreement, or when the product has been out-licensed, the responsibility for disclosure may be dependent on the agreement between parties. Under these circumstances, Merck KGaA will endeavor to gain agreement to share data in response to requests.

## Supplementary Materials

The supporting information can be downloaded at: https://www.mdpi.com/article/10.3390/ijms24098040/s1.

## Glossary

Four-parameter logistic equation	Also referred to as a dose–response curve. The equation contains four parameters or variables related to the graphical properties of the curve: the bottom and top plateaus of the curve, the half maximal effective concentration (EC_50_) and the slope factor.
Antennarity	The number of branching glycans (bi-, tri- or tetra-antennarity) influences acidity and can impact the binding of the follicle-stimulating hormone receptor (FSHR). Indeed, bulky and extended glycans may result in a delayed receptor response while relatively smaller and more compact follicle-stimulating hormone (FSH) glycoforms (e.g., the bi-antennary at αAsn-52) have more rapid FSHR binding [11]. The higher the number of antennae, the greater the probability of having complete glycan moieties (i.e., with galactose and sialic acid attached). In fact, sialic acid can be added only if a galactose molecule is present in the carbohydrate chain of the terminal branch. Therefore, the number of antennae is indirectly positively correlated with FSH glycoform acidity through sialylation. Alternatively, the sequential addition of N-acetylgalactosamine (GalNAc) and sulfate produces sulfated oligosaccharides [27].
Biological activity	The specific ability or capacity of a product to achieve a defined biological effect. A valid biological assay to measure the biological activity should be provided by the manufacturer [7]. Biological activity depends on the assay used to measure it.
Biopotency/potency	The quantitative measure of biological activity based on the attributes of the product (e.g., the nature and quantity of product-related substances, product-related impurities and process-related impurities) that is linked to the relevant biological properties (e.g., biological activity). A valid biological assay (e.g., in vitro assay or in vivo animal assay or biochemical assays) to measure the biological activity (biopotency) should be provided by the manufacturer of the product. When a valid bioassay is not suitable to measure the biological activity, potency may refer to the quantity of protein (see definition of Quantity in this Glossary) [7].
Glycans (also called “oligosaccharides”)	Carbohydrate-based polymers that are essential for the structure, energy storage, receptor binding affinity and clearance of glycoproteins
Glycoforms	Variants (isoforms) of a glycoprotein that have different glycans attached, and/or are glycosylated at different amino acid residues [11].
Glycoproteins	Proteins that contain glycans covalently attached to amino acid side-chains. This post-translational process occurs in the cell’s endoplasmic reticulum (ER) and Golgi apparatus, and is known as “glycosylation” [37].
Glycosylation	The addition of glycans (*N*- and *O*-glycans) to proteins. It is a highly regulated mechanism of secondary protein processing within cells, and affects the three-dimensional configuration of proteins, as well as their function and stability. Glycosylation is critical for the action of glycoproteins as well as for their pharmacokinetics and pharmacodynamics (PK/PD) [11].
Inflection point	A point on a curve at which the sign of the curvature changes.
In-process control (test)	In-process tests are tests which may be performed during the manufacture of either the drug substance or drug product, rather than as part of the formal battery of tests which are conducted prior to release [7,38].
International standard	The World Health Organization (WHO) international standards and reference materials provide a common set of standards that are used to ensure the quality of biological medicines worldwide. Currently, the reference standard to measure FSH biopotency is follitropin alfa [39].
International Unit (IU)	For each substance, there is international agreement on the biological effect that is expected for 1 IU based on the potency of a reference standard (see also Biopotency/Potency) [40].
International IU of FSH	The activity contained in a stated amount of the International Standard (IS) of follitropin-alfa [15]. The equivalence in IU of the IS is stated by the WHO for any new certified IS [41]. The first WHO IS defined 1 IU as the amount of FSH exerting an activity corresponding to 0.11388 mg of pure Human Urinary FSH [16,42]. Each new standard is calibrated and validated according to the WHO Expert Committee of International Standard. The replacement IU is defined by the contents of the ampoule of the new standard. Every effort is made to maintain the continuity of the IU, but the replacement IU is not formally traceable to the first IS [41].
Parallel Line Analysis (PLA) software	A statistical test for parallelism. Commonly used to compare dose–response curves to estimate the potency of a test substance relative to the potency of a standard.
Pharmacodynamics	Study of pharmacological actions on living systems, including the reactions with and binding to cell constituents, and the biochemical and physiological consequences of these actions [43].
Pharmacokinetics	The activity of drugs in the body over a period of time, including the processes by which drugs are absorbed, distributed in the body, localized in the tissues, metabolized and excreted [40].
Post-translational modifications	The enzyme-mediated covalent addition of functional groups to proteins during or after synthesis that increase the functional diversity of the protein [44,45]. Can include the oxidation of methionine residues. The methionine residues in FSH are not directly located in regions that are critical for binding to the FSHR: methionine 29 is involved in α-β subunit heterodimerization; methionine 47 is located close to the FSHR binding site, but it is not directly involved in the ligand–receptor interaction; methionine 71 is located close to the heterodimerization site but is not directly involved in heterodimerization; and methionine 109 is located in the non-structural C-terminal region. However, the oxidation of these residues may lead to conformational changes, with the potential for indirect effects on biological activity, PK or protein aggregation, with the alteration of the immunogenicity of therapeutic proteins [23].
Quality control	The process used to ensure that all preliminary testing is carried out and that the quality of the final product has been deemed to meet all standards of identity, strength, quality and purity [7,38].
Quantity	The physicochemical measurement of protein content. If physicochemical tests alone are used to quantitate the biological activity (based on the correlation between the expected clinical response and the activity in the biological assay, as established in pharmacodynamic or clinical studies), potency should be expressed in mass [7].
Reference House Standard (RHS)	Biotech material, appropriately characterized, produced from validated production processes and used as reference standard. It is characterized and calibrated versus a suitable IS using an appropriate characterization panel and bioassay. The RHS is intended for use in specified analytical tests, in which its properties are compared with properties of samples under examination.
Relative potency	Measured by comparing the concentration–response curves of a manufactured test batch with that of a RHS.
Sialylation	The covalent addition of sialic acid to the terminal end of oligosaccharides attached to the protein core. Terminal sialylation, namely the presence or absence of a sialic acid (a2,3 and/or a2,6 *N*-Acetyl neuraminic acid (Neu5Ac)) that caps glycan branches, is the major factor governing the in vivo clearance rate. More acidic glycoforms have longer elimination rates due to reduced renal clearance, while less acidic glycoforms have a higher affinity for the FSHR [2].
Specific activity (units of biological activity per mg of product)	The quantitative measure of biological activity evaluated as biological activity/mass and can also serve to measure the purity and consistency of a product.
