# Supplementary material for: Biological Assay to Determine Gonadotropin Potency: From In Vivo to In Vitro Sustainable Method
Source: Int J Mol Sci. 2023 Apr 28;24(9):8040. doi: 10.3390/ijms24098040 (PMC10178553; doi:10.3390/ijms24098040)

Supplementary Materials

Supplementary Figure S1: Example dose-response curves for acid enriched variants versus RHS analyzed using **A**) *in-vitro* and **B**) *in-vivo* bioassay.

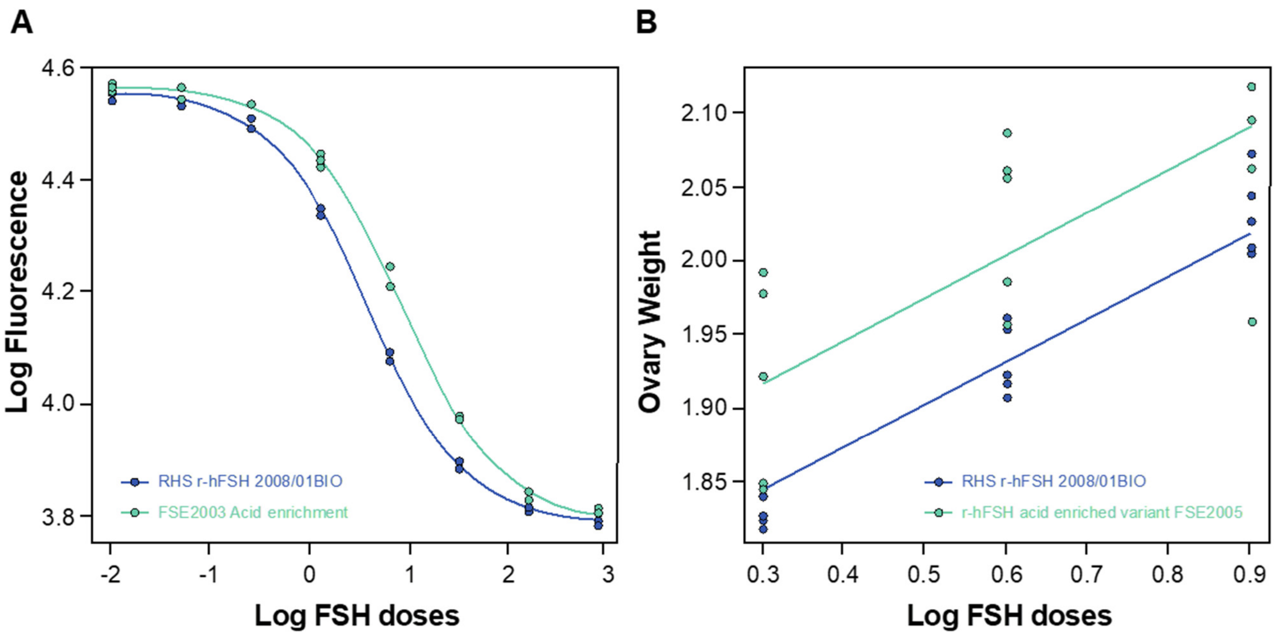

Supplementary Figure S2: ANOVA test for significance between untreated and acid enriched samples **A**) *in-vitro* and **B**) *in-vivo* assay specific activity.

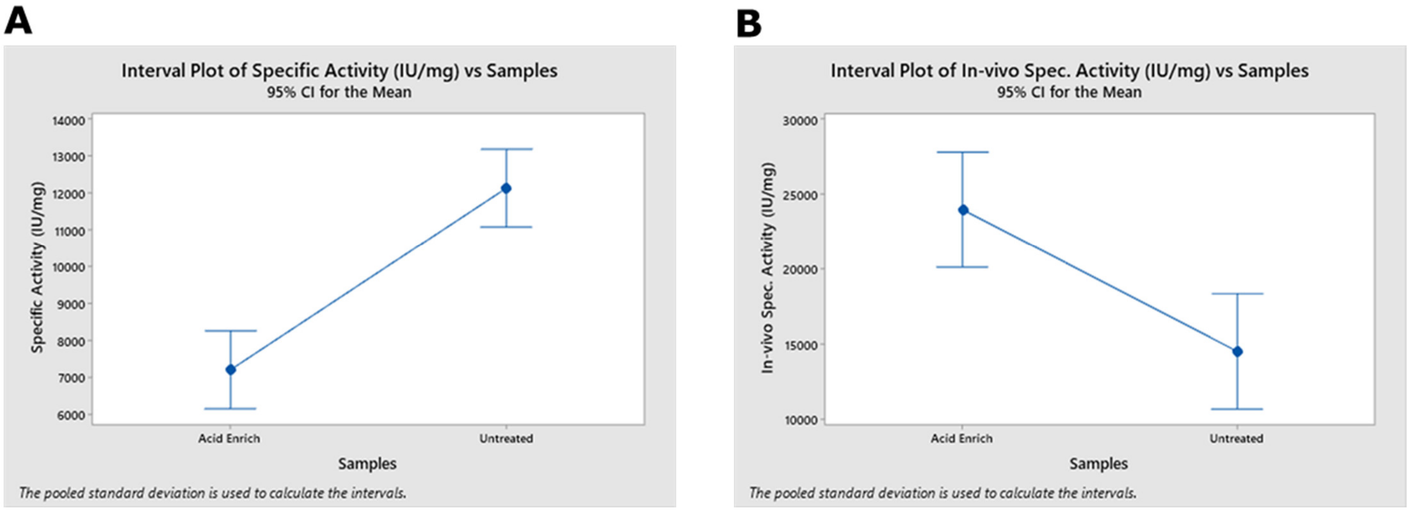

Supplementary Figure S3: Example dose-response curves for basic enriched variants versus RHS analyzed using **A**) *in-vitro* and **B**) *in-vivo* bioassay.

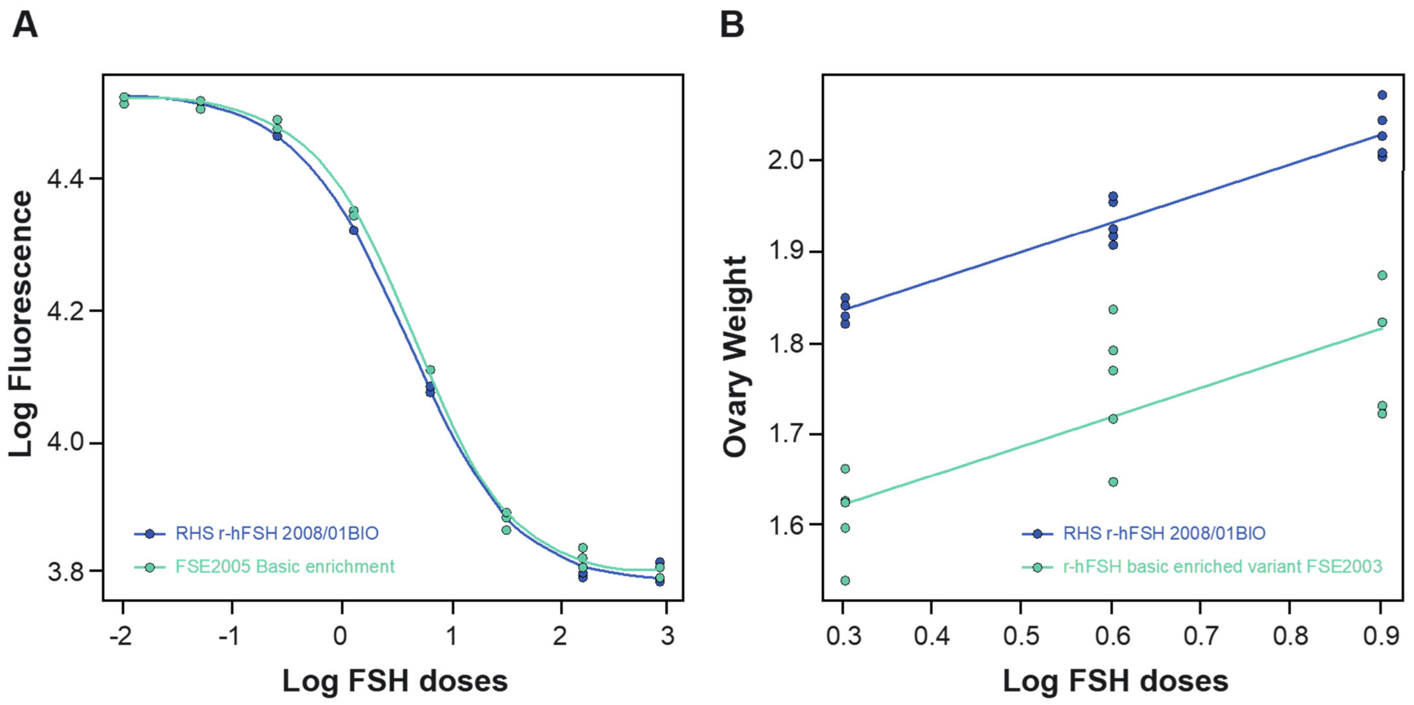

Supplementary Figure S4: ANOVA test for significance between untreated and basic enriched samples **A**) *in-vitro* and **B**) *in-vivo* assay specific activity.

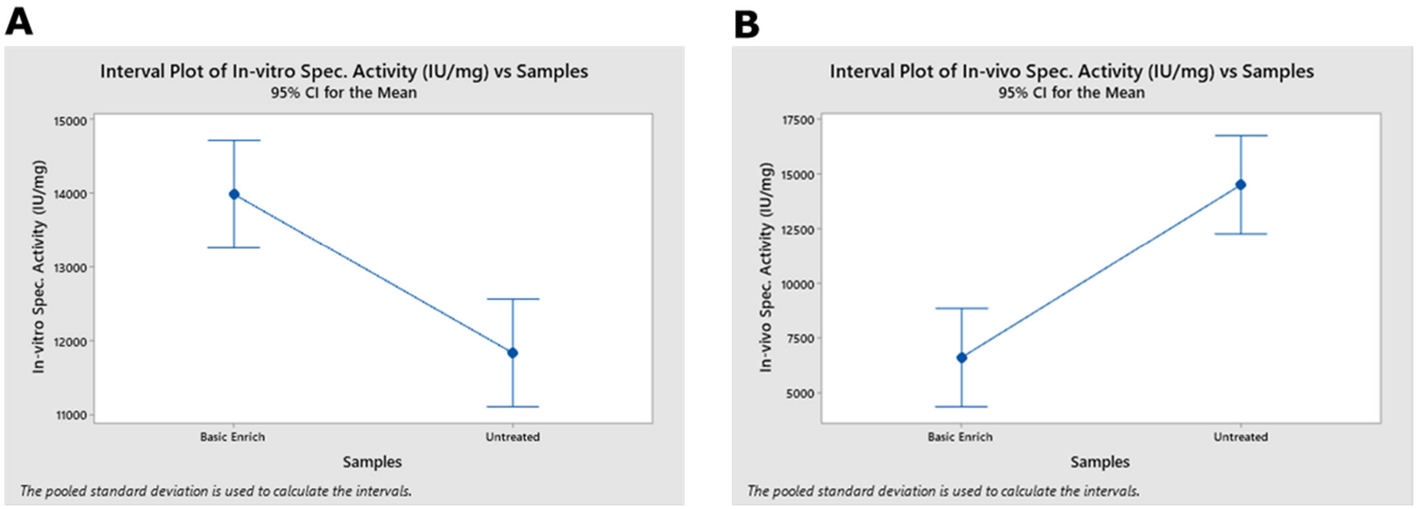

Supplementary Figure S5: Example dose-response curves for oxidized variants versus RHS analyzed using **A**) *in-vitro* and **B**) *in-vivo* bioassay.

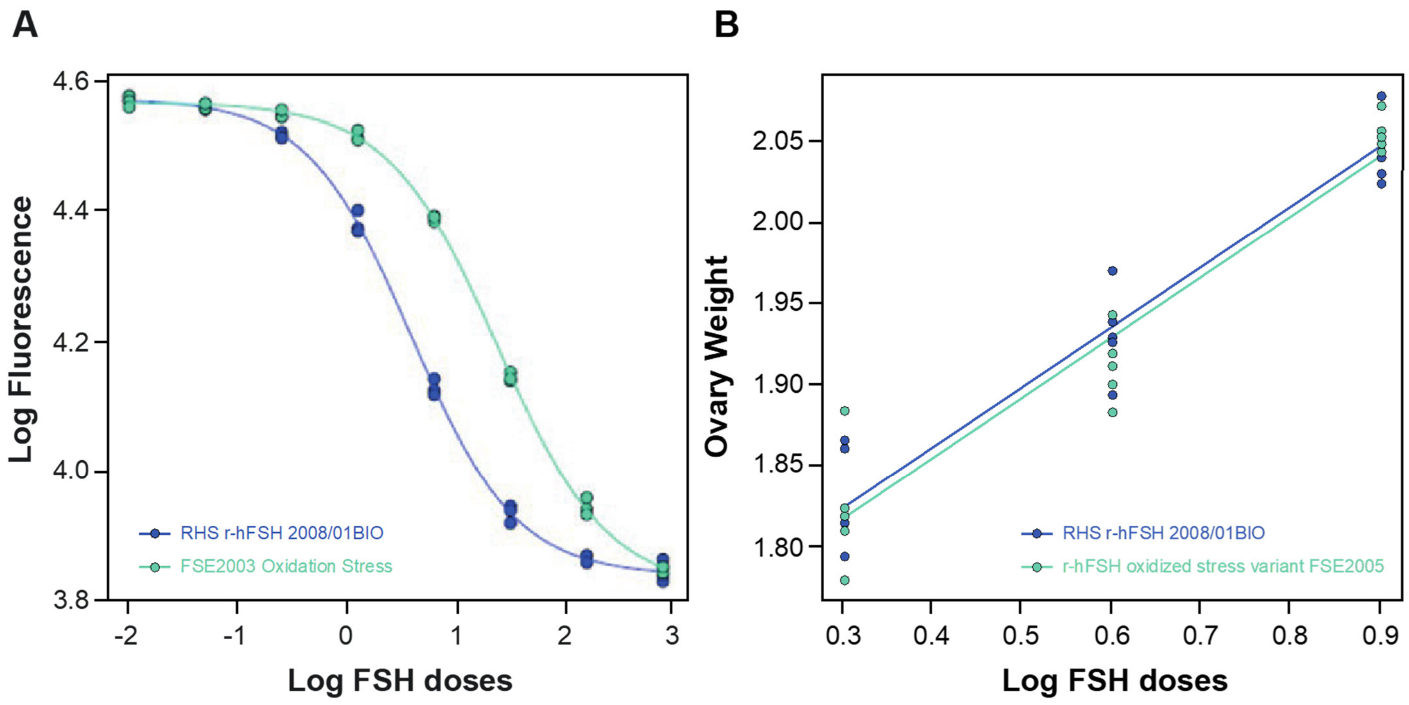

Supplementary Figure S6: ANOVA test for significance between untreated and oxidized samples **A**) *in-vitro* and **B**) *in-vivo* assay specific activity.

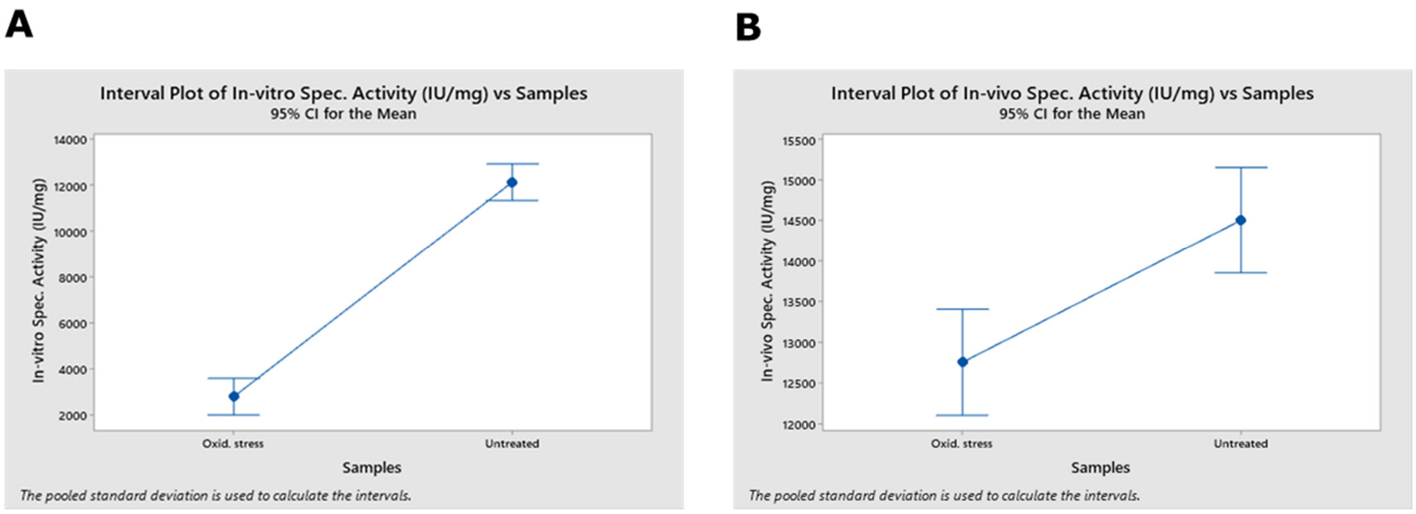

Supplementary Figure S7: Example dose-response curves for acid pH stressed variants versus RHS analyzed using **A**) *in-vitro* and **B**) *in-vivo* bioassay.

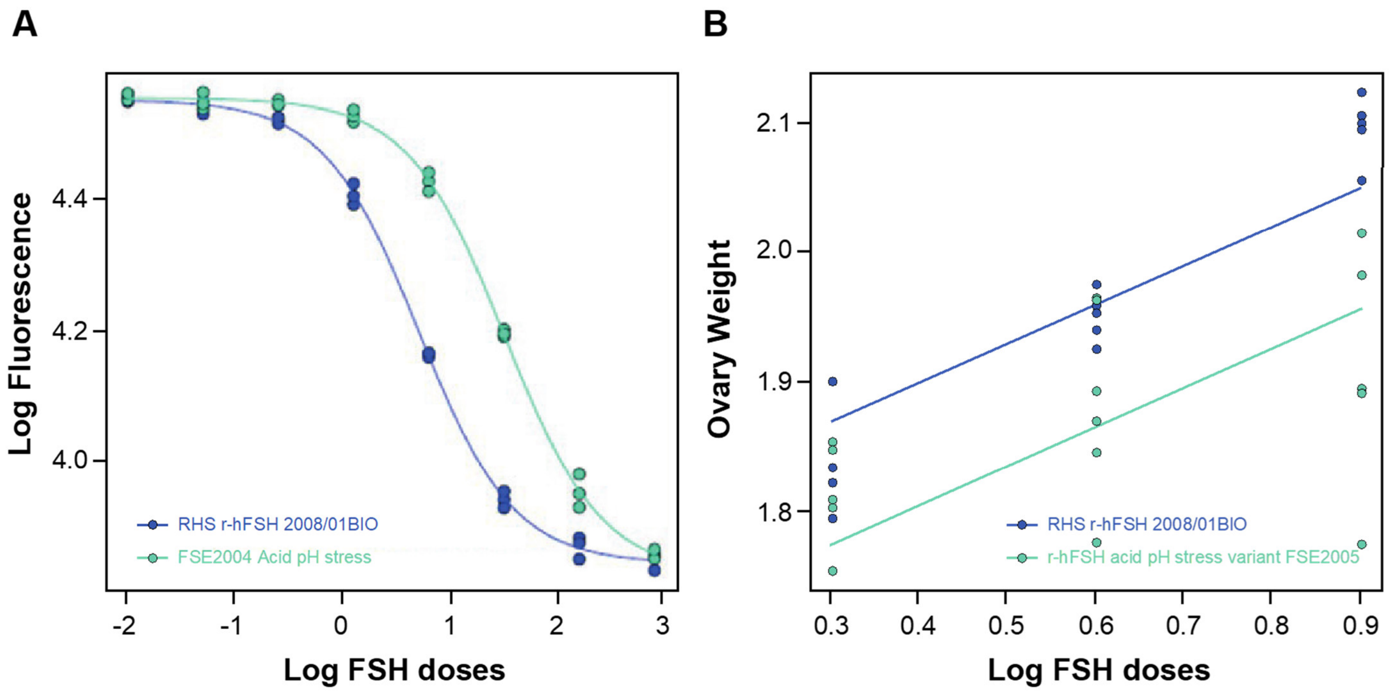

Supplementary Figure S8: ANOVA test for significance between untreated and acid pH stressed samples **A**) *in-vitro* and **B**) *in-vivo* assay specific activity.

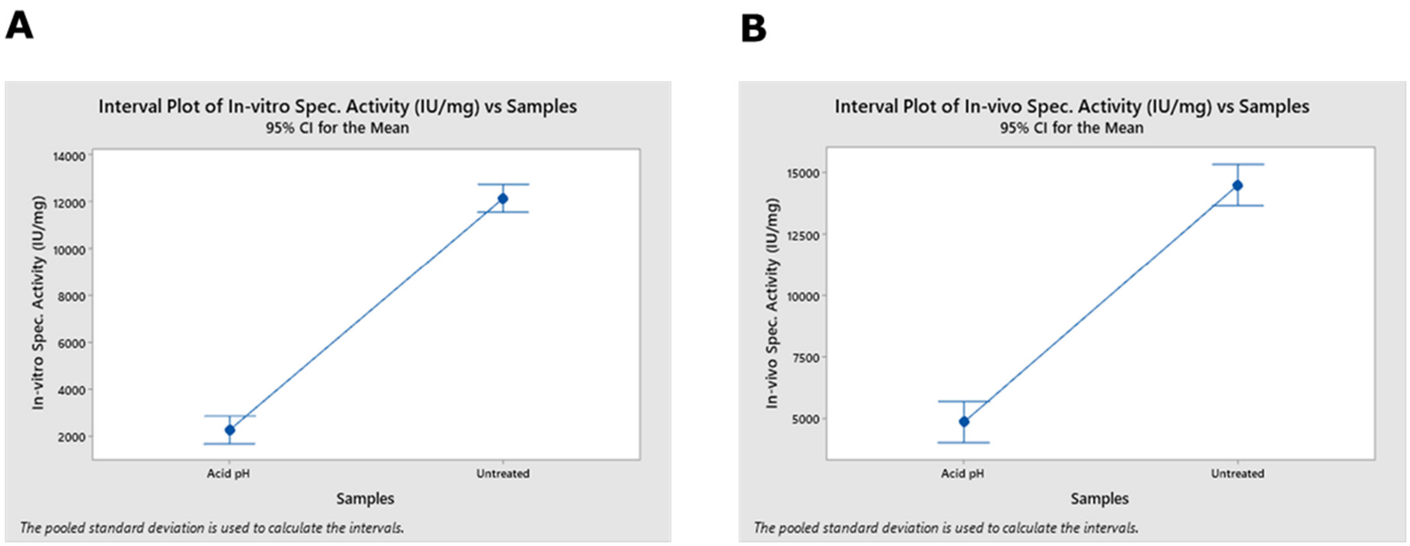

Supplementary Figure S9: Example dose-response curves for 100% desialylated FSE2012 **A**) totally desialylated and **B**) untreated FSE2012 versus RHS analyzed using the *in-vitro* bioassay and **C**) 100% desialylated FSE2012 versus RHS analyzed using the *in-vivo* bioassay.

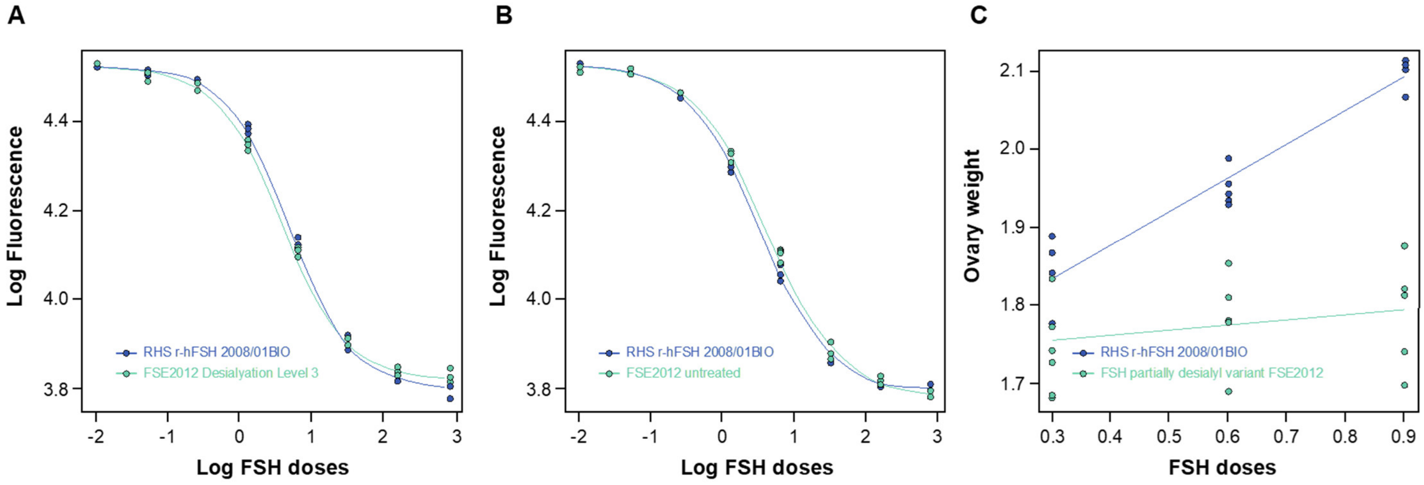

Supplementary Figure S10: ANOVA test for significance between untreated and totally desialylated samples **A**) *in-vitro* and **B**) *in-vivo* assay specific activity.

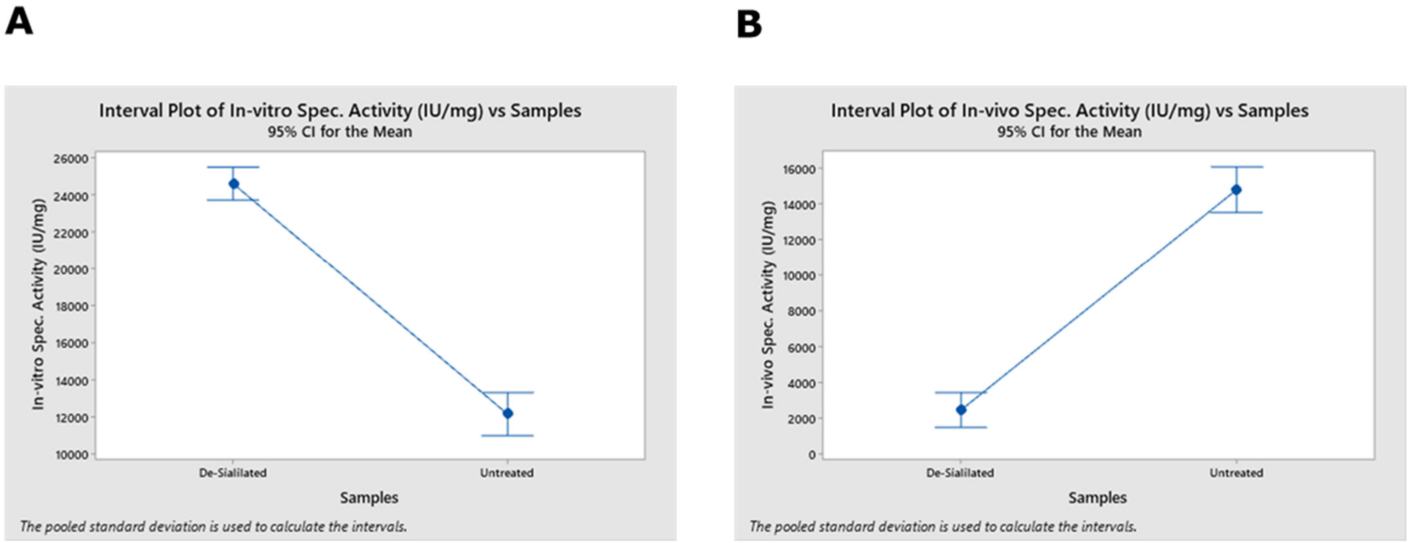

Supplementary Figure S11: ANOVA test for significance between untreated and desialylated and degalactosylated samples **A**) *in-vitro* and **B**) *in-vivo* assay specific activity.

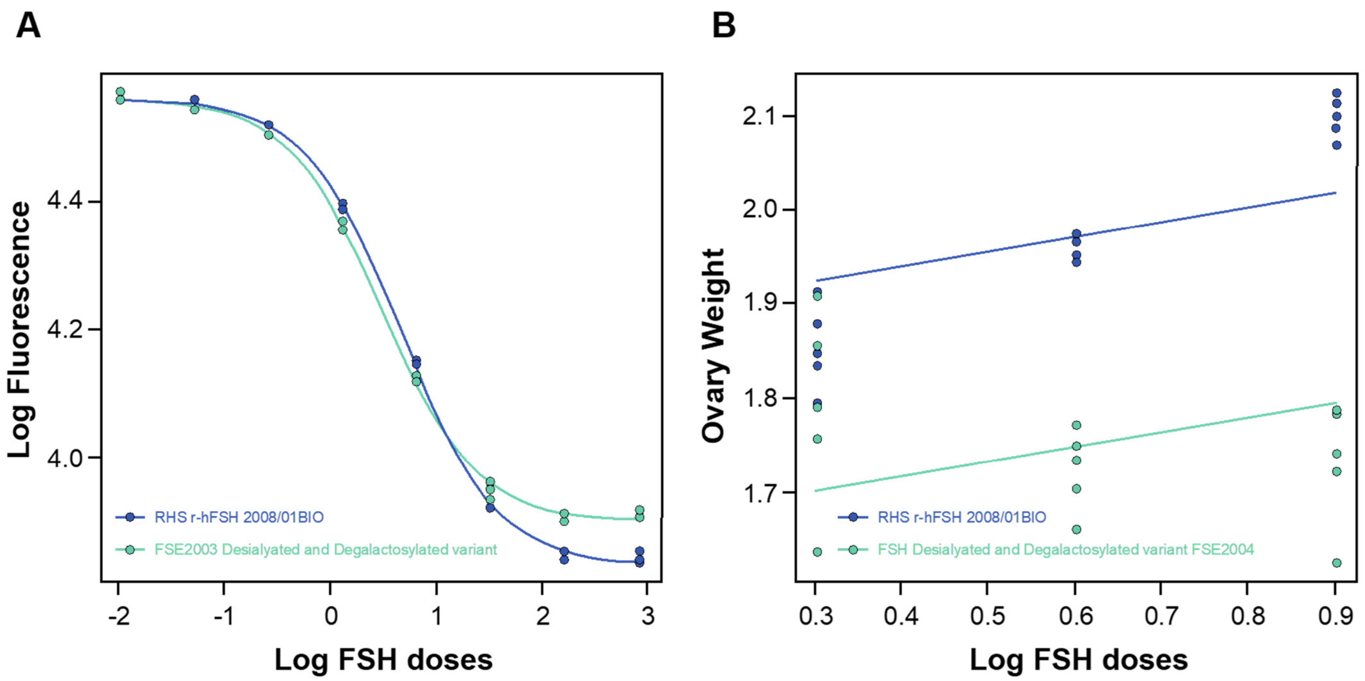

Supplementary Figure S12: Example dose-response curves for desialylated and degalactosylated variants versus RHS analyzed using **A**) *in-vitro* and **B**) *in-vivo* bioassay.

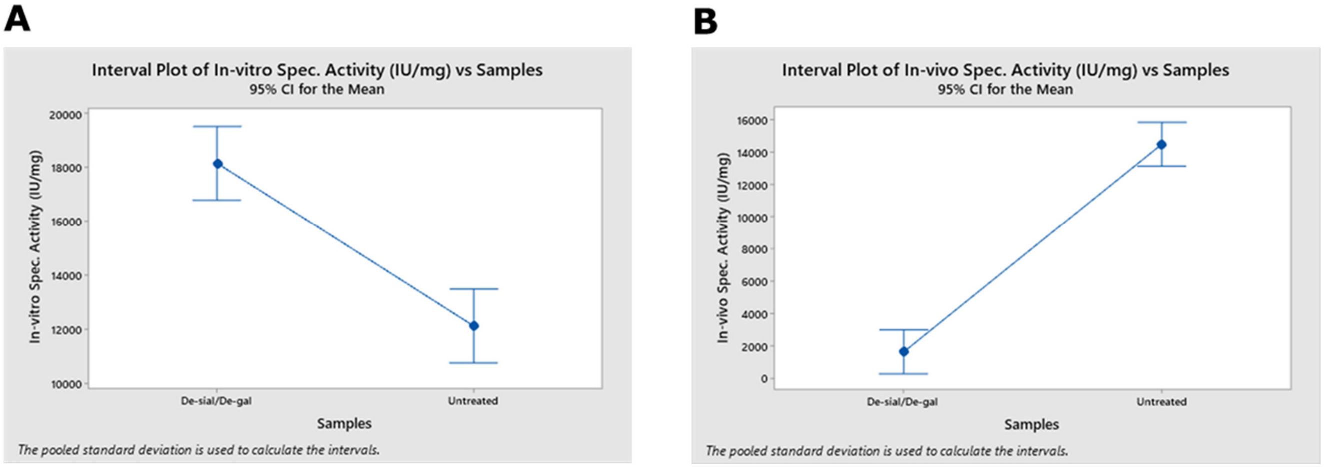

Supplement: Supplementary file 1 [file ijms-24-08040-s001.zip › ijms-2315672-supplementary.pdf]
